# The Inhibitory Effect and Mechanism of *Ferula akitschkensis* Volatile Oil on Gastric Cancer

**DOI:** 10.1155/2022/5092742

**Published:** 2022-03-29

**Authors:** Rong Han, Yun Sun, Ruoting Ma, Dexi Wang, Jianan Sun, Shengjun Zhao, Haiying Zhang

**Affiliations:** ^1^Department of Pharmacy, Affiliated Hospital of Traditional Chinese Medicine of Xinjiang Medical University, Urumqi 830000, Xinjiang, China; ^2^Xinjiang Key Laboratory of Processing and Research of Traditional Chinese Medicine, Urumqi 830000, Xinjiang, China; ^3^Department of Traditional Chinese Medicine, The Traditional Chinese Medicine College of Xinjiang Medical University, Urumqi 83000, Xinjiang, China; ^4^Graduate School, The Fourth Clinical College of Xinjiang Medical University, Urumqi 83000, Xinjiang, China; ^5^State Key Laboratory of Pathogenesis, Prevention and Treatment of High Incidence Diseases in Central Asia, Xinjiang Medical University, Urumqi 830000, Xinjiang, China

## Abstract

*Ferula akitschkensis* volatile oil (FAVO) has a good inhibitory activity on gastric cancer cell proliferation, but the mechanism of action is not yet clear. In this study, we tested the antigastric cancer efficacy and mechanism of FAVO using both *in vivo* and *in vitro* models. The results showed that FAVO effectively inhibited the proliferation, migration, and invasion of human gastric cancer SGC-7901 cells, the formation of small tubules of human umbilical vein endothelial cells as well as zebrafish intersegmental vessel and intestinal vein angiogenesis. *In vivo* experiments showed that FAVO significantly delayed the growth of SGC-7901 tumor-bearing nude mice and induced higher serum IL-2 and IFN-*γ* and reduced serum IL-6. Western blot results showed that FAVO reduced the expression of HIF-2*α*, VEGF, VEGFR2, P-VEGFR2, Akt, and P-Akt in SGC-7901 cells with CoCl_2_ induced hypoxia. We further clarified the main chemical components of FAVO through GC-MS analysis. In summary, FAVO may inhibit tumor growth and angiogenesis via inhibiting the HIF-2*α*/VEGF signaling pathway.

## 1. Introduction

*Ferula* belongs to Peucedaneae Drude of Apiaceae and is mainly distributed in Central Asia, including Iran, Pakistan, and Turkey. There are 31 species distributed in China [1]. *Ferula* has many biological activities such as anti-inflammation, anticancer, and antiangiogenesis [2–5]. For example, it has been reported that *Ferula* inhibited the proliferation and metastasis of human colon cancer HCT116 cells, human glioma U87 cells, Raji lymphoma cells, cervical adenocarcinoma HeLa cells, and breast cancer MCF-7 cells and induced apoptosis as well as cell cycle arrest [4, 6–8]. Our previous results also showed the ethyl acetate extract of *Ferula* sinkiangensis had an inhibitory effect on colon cancer, gastric cancer, and cervical cancer [9–11].

The medicinal part of *Ferula akitschkensis* is root and oleoresin, which have been used to treat headaches, colds, stomach diseases, and other diseases [12–14]. *Ferula akitschkensis* can regulate estrogen activity and neutrophils and inhibit methicillin-resistant *Staphylococcus aureus*. *Ferula* plants are rich in volatile components, which are mostly extracted by steam distillation and solvent methods. *Ferula akitschkensis* volatile oil (FAVO) is the characteristic active component of *Ferula akitschkensis* and has antitumor, antibacterial, anti-inflammatory, antioxidative, and antiparasitic effects. For example, Sheng et al. [15] reported that FAVO prepared by steam distillation and microwave extraction had a strong inhibitory effect on gastric cancer cell SGC-7901. Hosseinzadeh et al. [16] found that FAVO had cytotoxicity on colon cancer CT26 cells and could induce apoptosis. Daneshkazemi et al. [17] showed that FAVO had antibacterial activity against 4 kinds of oral bacteria (*Streptococcus mutans*, *Streptococcus sanguis*, *Streptococcus salivarius*, and *Lactobacillus rhamnosus*). Youssef et al. [18] reported that FAVO had strong antioxidant activity. The composition of FAVO varies greatly among different species of *Ferula akitschkensis*. The main components of FAVO are mainly terpenes and alkenes, and some FAVO also has polysulfides. However, the studies about FAVO on gastric cancer are less reported. Additionally, the mechanism and effective chemical components of FAVO in inhibiting human gastric cancer are still unclear, which all further limits its clinical application.

The rapid proliferation of tumor cells causes the tissue within the tumor to be under hypoxia due to lack of blood supply. Hypoxia not only induces the differentiation and proliferation of vascular endothelial cells but also promotes tumor angiogenesis [19, 20]. Hypoxia inducible factor (HIF), including HIF-1*α* and HIF-2*α*, produced by tumor cells is involved in the signal transduction between cells. HIF-2*α* has 48% sequence homology with HIF-1*α*. HIF-1*α* is activated during short-term hypoxia, which will gradually decrease or even disappear under continuous hypoxia. The current research mainly focuses on HIF-2*α*. HIF-2*α* continues to increase during hypoxia and regulates the expression of hypoxia response genes under continuous hypoxia, such as VEGF [21–23]. VEGF, also known as vascular permeability factor, plays a vital role in the occurrence, development, invasion, and metastasis of tumors [24–27]. VEGF binds to receptors on the cell membrane, promotes the proliferation, differentiation, and migration of vascular endothelial cells, and promotes the permeability of vascular endothelial cells [28–30]. VEGFR2 (vascular endothelial growth factor receptor 2), the main receptor of VEGF, is mainly distributed on the surface of vascular endothelial cells, lymphatic endothelial cells, megakaryocytes, and hematopoietic stem cells and is the most important factor for VEGF to regulate angiogenesis [31, 32].

In this study, we investigated the effects and mechanisms of FAVO on the proliferation, migration, and invasion of human gastric cancer SGC-7901 cells. The chemical components of FAVO were primarily determined by GC-MS. Both in vitro and in vivo experiments were performed. Our results confirm that FAVO could affect the proliferation, migration, and invasion of human gastric cancer cells via the HIF-2*α*/VEGF pathway. Our results demonstrate that *Ferula akitschkensis* may serve as a potential antitumor drug and provide a new option for combination therapy or supportive treatment of gastric cancer.

## 2. Materials and Methods

### 2.1. Extraction of FAVO

The root of *Ferula akitschkensis*, which was collected from Jimunai County, Altay Region, Xinjiang, China in June 2019 and was identified by Yonghe Li, the Chief Pharmacist of traditional Chinese medicine from the Chinese Medicine Hospital Affiliated to Xinjiang Medical University, was chopped into small pieces. After that, 10 times the amount of water were added, and the samples were placed in an electric heating mantle connected with the volatile oil tester and the reflux condenser. The samples were slowly heated to boiling and kept boiling for 5 h. After drying with anhydrous sodium sulfate, an oily liquid with a special fragrance was obtained.

### 2.2. GC-MS Analysis

GC-MS analysis was performed on 5977A MSD-7890B GC/MS (Agilent, USA). Briefly, for gas chromatographic analysis, a chromatographic column HP-5MS capillary column (30 m × 0.25 mm × 0.25 *μ*m) (Agilent, USA) was used. The injection volume was 0.05 *μ*L, and the split ratio was 60 : 1. The program was as follows: starting temperature 60°C, 2 min; heating up to 80°C at a rate of 4°C/min, 5 min; heating up to 180°C at 2°C/min, 5 min; heating up to 200°C at 10°C/min, 2 min. Mass spectrometry conditions are as follows: ionization method, EI; ionization energy, 70 eV; ion source generator temperature, 230°C; and mass scanning range, 30–350°amu.

### 2.3. MTT Assay

Human gastric cancer SGC-7901 cells, from the Institute of Cell Research, Chinese Academy of Sciences (Shanghai, China), were inoculated into 96-well plates, and FAVO of different concentrations (300 *μ*g mL^−1^, 150 *μ*g mL^−1^, 75 *μ*g mL^−1^, 37.5 *μ*g mL^−1^, 18.75 *μ*g mL^−1^, and 9.375 *μ*g mL^−1^) was added for treatment for 24 h. After that, MTT (Sigma) was added and incubated for 3 to 4 h. Finally, 150 *μ*L DMSO was added for development. The absorbance (490 nm) was detected with a microplate reader (Thermo, USA). The proliferation inhibition rate (%)°=°(OD (control group)−OD (volatile oil group))/(OD (control group)−OD (blank group)) × 100%.

### 2.4. Transwell Assay

Gastric cancer SGC-7901 cells were seeded in a 6-well plate, and FAVO (37.5 *μ*g mL^−1^, 18.75 *μ*g mL^−1^, and 9.375 *μ*g mL^−1^) and cisplatin (30 *μ*g mL^−1^, Jiangsu Hansoh Pharmaceutical Group Co., Ltd., China) were added. After treatment for 48 h, SGC-7901 cells were collected, resuspended in serum-free cell culture medium, and added to the upper chamber of the Transwell chamber (Corning, USA). The chamber precoated with Matrigel (Corning) was used for the invasion assay and that without Matrigel was used for the migration assay. Cell culture medium containing 10% fetal bovine serum was added to the lower chamber. After culturing for 48 h, the cells in the lower Transwell chamber were fixed with paraformaldehyde (4%) for 30 min and stained for 30 min. Finally, the number of migrated and invaded cells in each group was counted under the microscope.

### 2.5. Zebrafish Intersegmental Vessel Angiogenesis

The vascular fluorescence transgenic zebrafish (VEGFR2 : GFP) was provided by the Scientific Research Laboratory of Longhua Hospital (Shanghai). The 1 *μ*g mL^−1^ Pronase E (SOLARBIO) was used to remove the egg membrane when the fertilized egg developed for 24 h. The zebrafish embryos were selected and transferred to a 24-well plate. FAVO was prepared with dimethyl sulfoxide (DMSO) to final concentrations of 37.5, 18.75, and 9.375 *μ*g mL^−1^. The normal control group was treated with culture medium containing 0.1% DMSO. After FAVO treatment, the sample was placed in a light incubator at 28°C for 14 h, and then, the number of zebrafish intersegmental vessels was observed and calculated by a fluorescence microscope (IX71-12FL/PH; Olympus).

### 2.6. Angiogenesis of the Inferior Intestinal Vein of SGC-7901-Transplanted Zebrafish

The newborn zebrafish embryos were lightly anesthetized with 1% tricaine. Then, the human gastric cancer SGC-7901 cells adjusted to 2 × 106 mL^−1^ (about 0.1 *μ*L, about 200 cancer cells) were injected under the yolk of the embryo with a microinjector. Afterwards, the zebrafish was treated with FAVO (37.5, 18.75, and 9.375 *μ*g mL^−1^) and cultured at 28°C for 48 h. After that, the anesthetized embryos were placed under a confocal microscope (FV-1000, Olympus) to analyze the effects of FAVO on the subintestinal veins (SIVs) of zebrafish.

### 2.7. Endothelial Cell Tube Formation Assay

Human umbilical vein endothelial cells (HUVECs), from the Institute of Cell Research, Chinese Academy of Sciences (Shanghai, China), were placed on a 96-well plate precoated with Matrigel. Then, HUVECs were suspended in 50 *µ*L basal F-12K medium (2.5 × 105 cells/mL) that also contained VEGF (50 ng/mL) and treated with or without various concentrations of FAVO (37.5, 18.75, and 9.375 *μ*g mL^−1^) for 8 hours. Through the length of tubules and the number of branch points per unit area, the effect of FAVO on the ability of HUVECs to form tubules was evaluated.

### 2.8. Animals

The BALB/c nude mice (weighing 18–22 g) were obtained from Vital River Laboratory Animal Technology Co., Ltd. (Beijing, China). They were kept in standard conditions. All animal experimental procedures were approved by the Ethics Review Board of The First Affiliated Hospital of Xinjiang Medical University.

### 2.9. Tumor Bearing Model Establishment and Treatment and Grouping of Animals

Nude mice model bearing gastric cancer SGC-7901 transplanted tumor was established by inoculating of 0.2 mL of 1 × 107 cells mL^−1^ SGC-7901 under the axilla of the left forelimb. On the next day, mice (10 mice in each group) were randomly divided into control group, tumor-bearing model group, cisplatin group, and FAVO (low, medium, and high dose) groups. The FAVO was given by gavage for 2 weeks. The low, medium, and high doses of FAVO were 0.75 g kg^−1^, 1.5 g kg^−1^, and 3.0 g kg^−1^, respectively. The dosage of cisplatin was 5 mg·kg^−1^, which was given intraperitoneally once every 3 days for two weeks.

### 2.10. Data Collection and Sampling

From the 7th day of administration, the length, width, and thickness of the transplanted tumor were measured, and the tumor volume was calculated as *π* × *a* × *b* × *c*/6 (a: tumor length, b: tumor width, c: tumor thickness). One hour after the last administration, mice were sacrificed after anesthesia. Blood samples were collected. The tumors were isolated and weighted. Tumor inhibition rate was calculated as

(1) $$\begin{array}{c} \frac{\text{Average tumor weight of type group}-\text{average tumor weight of drug}-\text{administered group}}{\text{Average tumor weight in model group}}\times 100\text{\%.} \end{array}$$

### 2.11. HE Staining

After 24 hours of fixation, the tumor tissues were dehydrated by ethanol and transparent with xylene before embedding in paraffin. After that, the samples were cut into sections and stained with hematoxylin and eosin. After mounting, the samples were observed under an optical microscope.

### 2.12. ELISA

The serum levels of IL-2, IFN-*γ*, and IL-6 in each group were detected by IL-2/IFN-*γ*/IL-6 ELISA kits (ExCell bio, Shanghai, China), respectively. The absorbance was analyzed with a microplate reader (xMarkTM, Bio-Rad). Finally, the serum levels of IL-2, IFN-*γ*, and IL-6 were calculated according to the standard curves.

### 2.13. Western Blot

According to different treatments, the human gastric cancer SGC-7901 cells were divided into normoxia group, hypoxia group, hypoxia + FAVO high/medium/low dose groups, and hypoxia + HIF-2*α* inhibitor PT2385 group. Cells in the normoxia group were cultured under normoxia. Hypoxia was induced with 200 *μ*mol L^−1^ CoCl_2_ (Sigma, USA) for 24 h. Then, for cells in hypoxia + FAVO high-/medium-/low-dose groups, FAVO (37.5, 18.75, and 9.375 *μ*g mL^−1^) were added for incubation of 24 h. For cells in the hypoxia + HIF-2*α* inhibitor PT2385 group, 5 *μ*mol·L^−1^ of HIF-2*α* inhibitor PT2385 (Selleck, USA) was added for incubation of 24 h. After treatment, the total protein of cells was extracted, and the protein concentration was determined by the BCA method. After SDS-PAGE electrophoresis, proteins were transferred to membrane and then subjected to labeling with primary and secondary antibodies. The primary antibodies from Cell Signaling Technology (Beverly, MA, USA) included anti-HIF-2*α* (#7096); anti-VEGF (#2463S); anti-VEGFR2 (#9698S); anti-P-VEGFR2 (#2478S); anti-AKT (#9272S); and anti-P-AKT (#4060S). The anti-*β*-actin primary antibody was purchased from Abcam (ab8226, USA). The secondary antibodies (#31430, Pierce Goat Anti-Mouse IgG (H + L), Peroxidase Conjugated, Thermo Scientific; #31460, Pierce Goat Anti-Rabbit IgG (H + L), Peroxidase Conjugated, Thermo Scientific) were used. After color development, ChemiScope mini chemiluminescence imaging system (Chemiscope 3000, Clinx Science Instruments Co., Ltd, Shanghai, China) was used to detect protein bands.

### 2.14. Statistical Analysis

The SPSS17.0 statistical software was used, and the data are expressed as mean ± standard deviation (SD). One-way ANOVA was used for multiple comparisons followed by pairwise comparison with the LSD method. A *P* value < 0.05 indicates that the difference is statistically significant.

## 3. Results

### 3.1. GC-MS Analysis of FAVO Components

**Figure 1:**
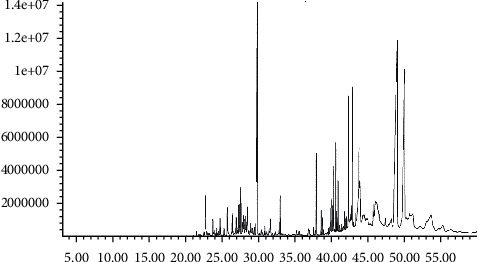
GC-MS fingerprint of the volatile oil of *Ferula akitschkensis*.

By referring to the standard database (NIST 98 Mass Spectral Library), the standard spectrum, and GC-MS analysis, we determined the components of FAVO by the peak area normalization method (Figure 1 and Table 1). The results showed that the volatile oil of *Ferula akitschkensis* was mainly composed of tricyclo [4.4.0.0(2,7)] dec-3-ene-3-methanol, 1-methyl-8-(1-methylethyl) (24.56%), and adamantane, 2-hydroperoxy-2-(2-oxiranyl) (13.86%).

### 3.2. FAVO Inhibits the Proliferation of Human Gastric Cancer SGC-7901 Cells

**Figure 2:**
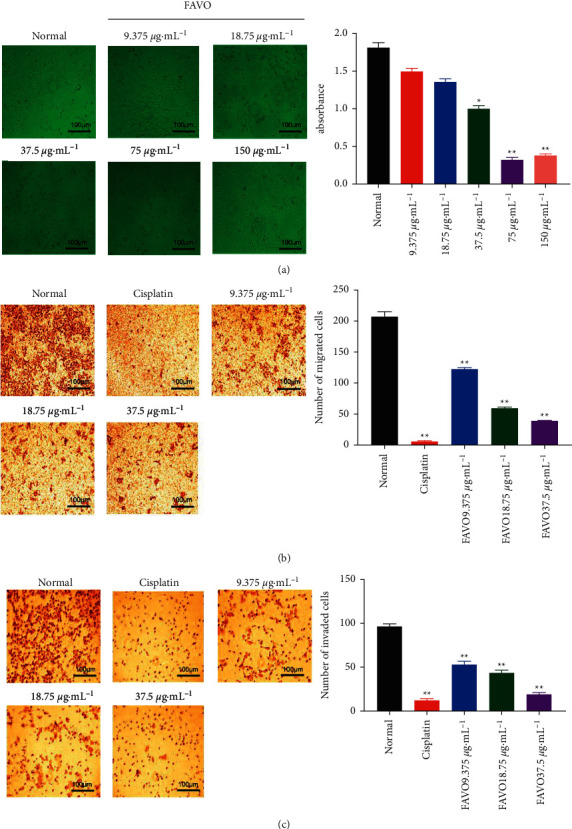
FAVO inhibits the proliferation, migration, and invasion of human gastric cancer SGC-7901 cells. SGC-7901 cells were treated with different concentrations of FAVO for 24 h. (a) The cell growth morphology of SGC-7901 cells were observed. Cell viability was detected by the MTT assay. The migration (b) and invasion (c) ability of the cells was tested by the Transwell assay. ^*∗∗*^*P* < 0.01, compared with normal control.

The MTT assay was performed to analyze cell proliferation. As shown in Figure 2(a), with the increase of the concentration, the growth of SGC-7901 cells was inhibited. The cell morphology changed from spindle to oval, and some cells were not attached to the culture plate and became suspended cells. The proliferation inhibition rate of cells treated with 75 *μ*g mL^−1^ and 150 *μ*g mL^−1^ FAVO was 99.17% and 95.89%, respectively, significantly lower than that of the normal group. According to the proliferation inhibition rate of each concentration, the IC50 of FAVO was determined at 20.33 *μ*g mL^−1^. The above results indicate that FAVO could inhibit the proliferation of human gastric cancer cells.

### 3.3. FAVO Inhibits Migration and Invasion of Human Gastric Cancer SGC-7901 Cells

Transwell assay tested the effects of different concentrations of FAVO on the migration and invasion of human gastric cancer SGC-7901 cells. The results showed that after 48 h of treatment with FAVO, the numbers of migrated (Figure 2(b)) and invaded (Figure 2(c)) cells were significantly decreased compared with the normal control group (*P* < 0.05). These results indicate that FAVO effectively inhibits tumor cell migration and invasion.

### 3.4. FAVO Inhibits Intersegmental Vessel Angiogenesis in Zebrafish

**Figure 3:**
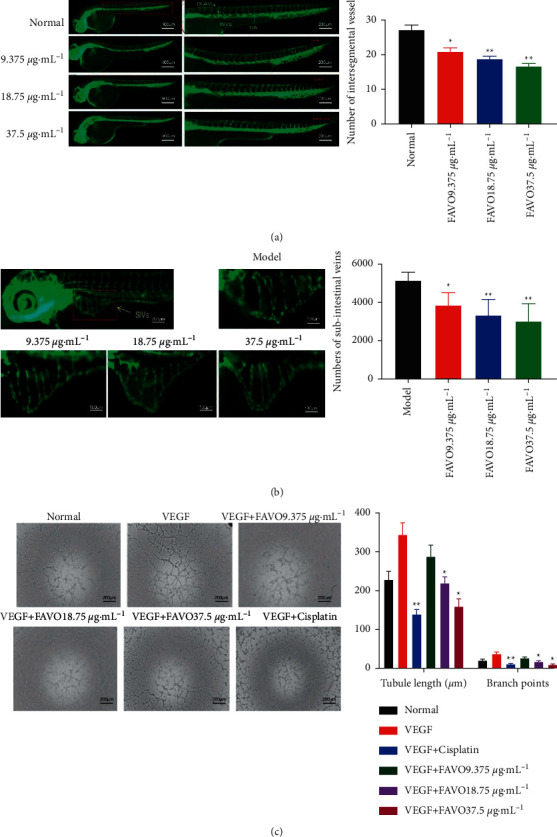
FAVO inhibits angiogenesis in zebrafish and human umbilical vein endothelial cells. (a) The effect of different concentrations of FAVO on the number of intersegmental vessels in zebrafish. (b) Human gastric cancer SGC-7901 cells were injected under the yolk of wild zebrafish embryos, and after treatment with different concentrations of FAVO for 48 h, the OD of the intestinal vein was analyzed by laser confocal microscopy. (c) After treating human umbilical vein endothelial cells with FAVO for 24 h, the cell growth morphology was observed. The length of the tubule per unit area and the number of branch points were measured. ^*∗*^*P* < 0.05, ^*∗∗*^*P* < 0.01, compared with the normal control.

The effect of FAVO on angiogenesis of the inferior intestinal vein was performed in SGC-7901 transplanted zebrafish. As shown in Figure 3(a), in the control group, the intersegmental vessels grew well and were evenly arranged. After treatment with FAVO, the fluorescence intensity of GFP was reduced. The vessels became thinner and partially missing. Statistically, the number of intersegmental vessels in zebrafish treated with FAVO was significantly reduced (*P* < 0.05). Thus, FAVO could inhibit intersegmental vessel angiogenesis in zebrafish.

### 3.5. FAVO Inhibits Angiogenesis in the Inferior Intestinal Vein of SGC-7901 Transplanted Zebrafish

Confocal microscopy showed that in the normal control group, the zebrafish embryonic SIVs were half-moon-shaped, fence-like, and showing continuous branches without collateral branches and budding (Figure 3(b)). After treatment with FAVO, the number of intestinal venous plexus, budding and collateral branches decreased dose-dependently. The OD value after FAVO treatment was significantly reduced (*P* < 0.05). The above results indicate that FAVO inhibits angiogenesis in the subintestinal vessels of the zebrafish intestine.

### 3.6. FAVO Inhibits the Tubule Formation Ability of HUVECs

Tube formation assay was performed with HUVECs. Compared with the normal control group, the length of the tubule per unit area and the number of branch points of HUVECs after FAVO treatment were significantly reduced (Figure 3(c)). The above results indicate that FAVO inhibits the tubule formation ability of HUVECs.

### 3.7. FAVO Inhibits Tumor Growth In Vivo

**Figure 4:**
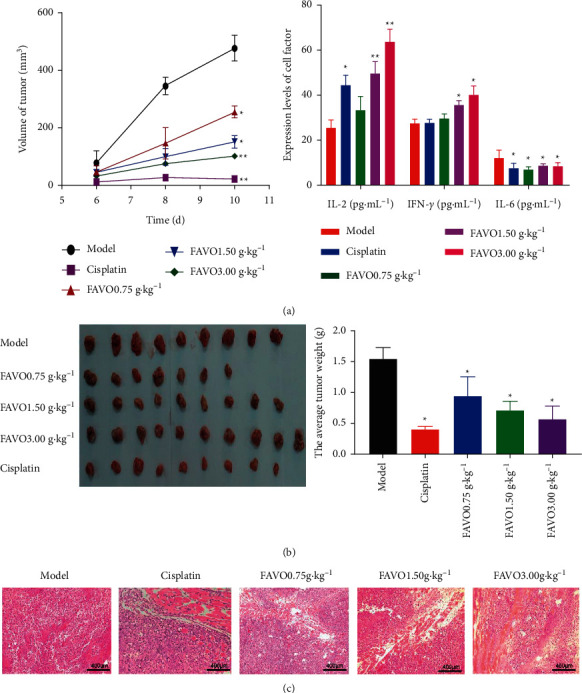
FAVO inhibits the growth of human gastric cancer SGC-7901 tumor in nude mice. After inoculation with human gastric cancer SGC-7901 cells, nude mice were given continuous gastric administration of FAVO (0.75 g kg^−1^, 1.5 g kg^−1^, and 3.0 g kg^−1^) for 2 weeks. (a) Tumor volume (right panel). After treatment for 2 weeks, the levels of IL-2, IFN-*γ*, and IL-6 in serum were detected by ELISA (left panel). (b) Tumor gross morphology and tumor weight after 2 weeks of intragastric administration. (c) HE staining of tumor. ^*∗*^*P* < 0.05, ^*∗∗*^*P* < 0.01, compared with the control group.

The effects of FAVO on tumor growth were further evaluated using nude mice bearing gastric cancer SGC-7901 transplanted tumor. Compared with the control group, the tumor growth of the nude mice treated with FAVO slowed down, with significantly decreased tumor volume (Figure 4(a)) and significantly lower tumor weight (Figure 4(b)). Thus, FAVO could inhibit tumor growth in vivo.

We further tested the levels of cytokines in the serum of tumor-bearing mice and found that FAVO increased the serum levels of IL-2 and IFN-*γ* but reduced the serum level of IL-6 (Figure 4(a)).

The pathological changes of tumors by HE staining showed that the tumor cells in the control group were densely arranged, with more cells in mitotic phases. In addition, there was avascular necrosis in some areas (Figure 4(c)). However, in the cisplatin treatment group, the pathological mitosis of the cells was reduced, but the infiltrating lymphocytes and macrophages increased. Correspondingly, after FAVO treatment, the number and mitosis of tumor cells decreased compared with the control group, but the infiltration of lymphocytes and macrophages increased. These results indicate that high-dose *Ferula akitschkensis* inhibited the tumor growth of SGC-7901 transplanted tumor nude mice.

### 3.8. Effect of FAVO on HIF-2*α*/VEGF Pathway

**Figure 5:**
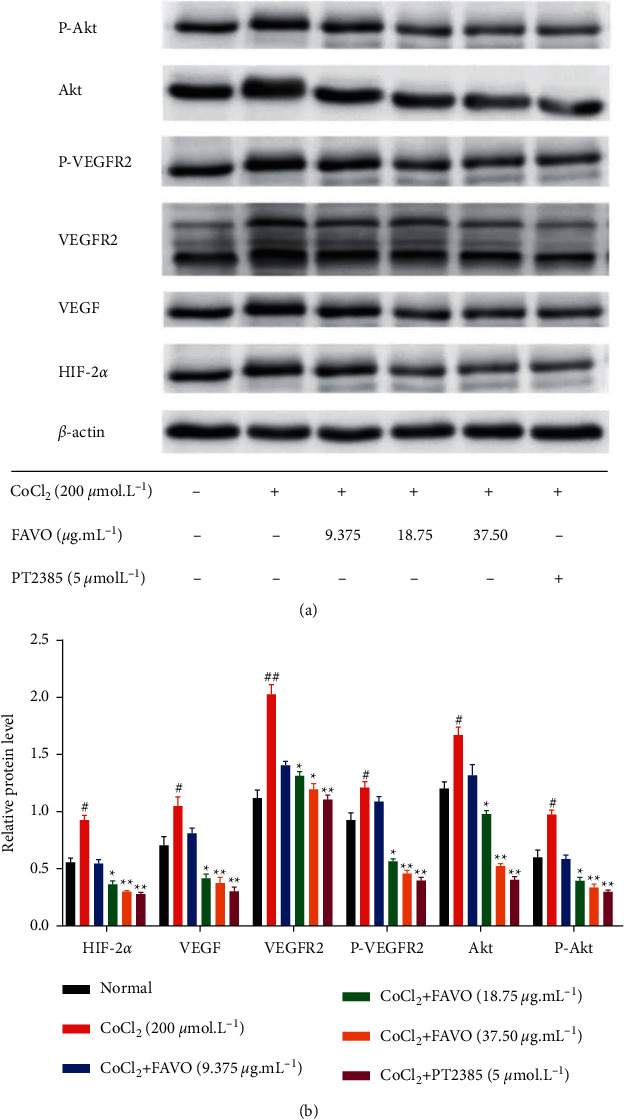
The effect of FAVO on the HIF-2*α*/VEGF pathway in gastric cancer SGC-7901 cells. The levels of HIF-2*α*, VEGF, VEGFR2, P-VEGFR2, P-Akt, and Akt in gastric cancer SGC-7901 cells after FAVO treatment detected by Western blot. (a) Representative western blot results. (b) Quantitative western blot results. ^#^*P* < 0.05^#^*P* < 0.05, ^##^*P* < 0.01, compared with the model control group; ^*∗*^*P* < 0.05, ^*∗∗*^*P* < 0.01, compared with the CoCl_2_ group.

Western blot analysis showed that under CoCl_2_-induced hypoxia, the expression levels of HIF-2*α* and downstream proteins including VEGF, VEGFR2, P-VEGFR2, P-Akt, and Akt were all significantly upregulated. However, after treatment with the HIF-2*α* inhibitor PT2385 and FAVO, their levels were all significantly reduced (Figure 5). Therefore, FAVO might inhibit the activation of the HIF-2*α*/VEGF pathway.

## 4. Discussion

Among *Ferula* plants, *Ferula akitschkensis* can be distinguished by smell [33]. The main components of the volatile oil of *Ferula akitschkensis* from Xinjiang and Fukang are sulfides and terpenes, which makes it to have a garlic-like odor [34]. Our analysis showed that components in FAVO contained no sulfides. The main components were tricyclo [4.4.0.0(2,7)] dec-3-ene-3-methanol, 1-methyl-8-(1-methylethyl) (24.56%), and adamantane, 2-hydroperoxy-2-(2-oxiranyl). However, further studies are needed to clarify the main effective components in FAVO.

Metastasis is a manifestation of malignant proliferation and development of tumors. Thus, we further determined the ability of FAVO in inhibiting the proliferation and migration of human gastric cancer SGC-7901 cells. The Transwell results showed that FAVO significantly inhibited the migration and invasion of human gastric cancer SGC-7901 cells. According to the “Guiding Principles of Pharmacodynamics of Anti-tumor Drugs,” the antitumor natural drugs should have a tumor-inhibiting rate greater than 40%. The SGC-7901 nude mouse model showed that the tumor inhibition rates of *Ferula akitschkensis* at low, medium, and high doses were 50.43%, 58.86%, and 63.20%, respectively, suggesting that *Ferula akitschkensis* is a potential anticancer drug. In addition, HE staining showed that the tumor cells in the tumor tissues of the *Ferula akitschkensis* high-dose group were loosely arranged and had more lymphocyte and macrophage infiltration. All these indicate the inhibitory effect of *Ferula akitschkensis* on SGC-7901 tumor cells.

Zebrafish is widely used in the screening of angiogenesis inhibitors, and in this study, we used zebrafish labeled with GFP-VEGFR2 [35, 36] for angiogenesis study. Due to the transparent characteristics of zebrafish embryos and specific expression of green fluorescent protein during the formation of blood vessels, the development of blood vessels can be observed more intuitively and finely. We found that FAVO significantly inhibited the formation of zebrafish intersegmental blood vessels. Moreover, it significantly inhibited tumor-induced intestinal vein angiogenesis in zebrafish transplanted with SGC-7901 cells. These suggest that FAVO may have antitumor effects by inhibiting tumor angiogenesis. The migration and fusion of endothelial cells and the formation of tubular structures are important links in the process of blood vessel formation, which may be blocked to inhibit tumor blood vessel formation [37]. Further results of this study showed that FAVO reduced the tubule length per unit area and the number of branch points, indicating that it inhibits the tubule-forming ability of HUVECs and has the effect of inhibiting tumor angiogenesis.

IL-2 is mainly produced by activated CD4^+^ T cells and CD8^+^ T cells, which can promote the survival of T cells and activate the growth of B cells. Studies have shown that IL-2 has a significant antitumor effect [38, 39]. IFN-*γ* has powerful immunomodulatory effect, which can extensively promote T and B cell differentiation and CTL maturation and stimulate B cells to secrete antibodies [40, 41]. IL-6 can be produced by T cells, macrophages, B cells and other cells. In the process of tumor development, IL-6, as a proinflammatory factor, has antiapoptosis effects, promotes cell proliferation, and blood vessel formation [42, 43]. IL-6 overexpression in cancer tissues promotes the growth and development of tumors by increasing the inflammatory response around the tissues in the tumors [44, 45]. In this study, we found that FAVO significantly promoted the expression of tumor IL-2 and IFN-*γ*, and inhibited the level of IL-6, which may be the mechanisms underlying the antitumor effects of FAVO. However, further studies are needed.

HIF-2*α* promotes angiogenesis during tumor growth, increases the stability of VEGF, and promotes the transcription and expression of VEGF-encoding genes [46]. VEGF is one of the most important factors to promote the proliferation and angiogenesis of vascular endothelial cells, and it plays a very important role in the process of angiogenesis [47]. VEGFR2 is a receptor tyrosine kinase that regulates the survival and proliferation of endothelial cells by activating PI3K/AKT signals [48–50]. The activation of AKT can further promote the survival of endothelial cells, induce angiogenesis, and promote tumor growth [51, 52]. In this study, we established a hypoxia model in vitro by CoCl_2_ treatment. The results showed that CoCl_2_ induced a significant increase in HIF-2*α* and its downstream proteins of VEGF, VEGFR2, P-VEGFR2, P-Akt, and Akt. The compound PT2385 binds to the PAS-B domain of HIF-2*α* subunit and affects the polymerization of HIF-2*α* subunit and *β*-subunit ARNT, thereby inhibiting the action of HIF-2*α* [53]. However, it has no effect on HIF-1. PT2385 reduced circulating VEGF-A levels and exhibited antitumor effects in a xenograft model [54]. PT2385 showed good efficacy in a phase I clinical trial (NCT02293980) in the treatment of advanced clear cell carcinoma of the kidney [55, 56]. Herein, after intervention with HIF-2*α* inhibitor PT2385, the levels of HIF-2*α* and downstream proteins were significantly reduced. Interestingly, similar results were obtained with FAVO. Therefore, we speculate that FAVO may abrogate the HIF-2*α*-VEGF-Akt signaling pathway, thereby inhibiting the proliferation and angiogenesis of human gastric cancer SGC-7901 cells.

## 5. Conclusions

In summary, FAVO weakened the proliferation, migration, and invasion of human gastric cancer SGC-7901 cells, inhibited vessel formation of zebrafish, impaired the tubule formation ability of HUVECs, suppressed tumor growth in vivo, increased the serum levels of IL-2 and IFN-*γ* in tumor-bearing nude mice, and inhibited HIF-2*α*-VEGF signaling. Thus, our results indicate that *Ferula akitschkensis* may become a potential antitumor drug, providing a new option for combination therapy or supportive treatment of gastric cancer.

## Figures and Tables

**Table 1:** Component analysis of volatile oil from *Ferula akitschkensis*.

No.	Retention time (min)	Compound	Area (Ab^*∗*^s)	Relative content (%)
1	22.751	(E)-*β*-Famesene	126051757	1.46
2	23.743	*β*-Vatirenene	43229897	0.50
3	24.729	(+)-*δ*-Cadinene	53738054	0.62
4	25.709	Froggatt ether	62168666	0.72
5	26.394	3,4-Pyridinediamine	61588463	0.71
6	26.925	Guaiol	46440842	0.54
7	27.285	Aristolene epoxide	122320760	1.41
8	27.539	Epi-7-*γ*-eudesmol	111028260	1.28
9	27.775	Ylangenol	39974833	0.46
10	27.887	*β*-Funebrene	54293770	0.63
11	27.987	Valencene	36060516	0.42
12	28.229	*α*-Muurolene	78498374	0.91
13	28.383	*α*-Gurjunene	27837705	0.32
14	28.466	*β*-Gurjunene	77109208	0.89
15	28.908	(8S, 14)-Cedran-diol	34477440	0.40
16	29.812	2-Hydroxy-2,4,4-trimethyl-3-(3-methylbuta-1,3-dienyl) cyclohexanone	1102247854	12.75
17	31.589	Isoshyobunone	47731042	0.55
18	32.946	Elemol	97480526	1.13
19	37.870	7-Cyclohexyl-2,3-dihydro-2-methyl-benzofuran	174053067	2.01
20	38.537	Ledene oxide-(II)	45863546	0.53
21	38.737	2-(4a,8-Dimethyl-2,3,4,4a,5,6-hexahydro-naphthalen-2-yl)-prop-2-en-1-ol	32056412	0.37
22	39.895	5-Methoxypsoralen	93722677	1.08
23	40.172	Linoleic acid ethyl ester	133054039	1.54
24	40.243	Ethyl Oleate	35037797	0.41
25	40.562	3b,4,5,6,7,7a,9,10,11,12-Decahydrobenzo[b]fluoranthene	176473713	2.04
26	40.845	[4ar-(4a*α*,6*α*,8a*β*)]-4a,5,6,7,8,8a-Hexahydro-6-[1-(hydroxymethyl) ethenyl]-4,8a-dimethyl-2(1H)-naphthalenone	80451285	0.93
27	41.730	3-Hydroxy-2,5,5,8a-tetramethyl-3,4,4a,5,6,7,8,8a-octahydronaphthalene-1-carboxylic acid, methyl ester	28793536	0.33
28	42.279	2-(3,7-Dimethylocta-2,6-dienyl)-Phenol	226236742	2.62
29	42.516	5-(7a-Isopropenyl-4,5-dimethyl-octahydroinden-4-yl)-3-methyl-penta-2,4-dien-1-ol	65737854	0.76
30	42.657	Murolan-3,9(11)-diene-10-peroxy	38467970	0.44
31	42.823	4a,7,7,10a-Tetramethyldodecahydrobenzo[f]chromen-3-ol	277813865	3.21
32	43.319	4,4-Dimethyl-(5*α*,17*β*)-androstan-17-ol	27798844	0.32
33	43.738	[3as-(3a*α*,6*β*,6a*α*,9a*β*,9b*α*)]-9a-[(acetyloxy)methyl] decahydro-6-methyl-3-methylene-azuleno [4,5-b] furan-2,9-dione	389145276	4.50
34	43.85	1-(9-Borabicyclo [3.3.1] non-9-yl)-3,5-bis(1,1-dimethylethyl)-4-ethyl-1H-pyrazole	198076654	2.29
35	44.334	3-Methyl-4-nitrobenzyl alcohol, n-butyl ether	67183486	0.78
36	45.774	o-Menth-8-ene	42185291	0.49
37	46.069	3′,4′-Dihydro-cholest-1-eno [2,1-a] naphthalene	194464417	2.25
38	48.124	2-(1H-Imidazo [4,5-b] pyridin-2-yl)-1-(4-morpholyl)-ethenone	75754222	0.88
39	48.944	1-Methyl-8-(1-methylethyl)-tricyclo [4.4.0.0(2,7)] dec-3-ene-3-methanol	2123603896	24.56
40	49.954	2-Hydroperoxy-2-(2-oxiranyl)-adamantane	1198660970	13.86
41	53.596	Betulinaldehyde	402406008	4.65

## Data Availability

The data used to support the findings of this study are included within the article.
